# Assessment of the availability and rationality of unregistered fixed dose drug combinations in Nepal: a multicenter cross-sectional study

**DOI:** 10.1186/s41256-017-0033-z

**Published:** 2017-05-08

**Authors:** Arjun Poudel, Mohamed Izham Mohamed Ibrahim, Pranaya Mishra, Subish Palaian

**Affiliations:** 1 Postdoctoral Research Associate, School of Clinical Sciences, Faculty of Health, Queensland University of Technology, Brisbane, Australia; 2 Department of Hospital and Clinical Pharmacy, Manipal Teaching Hospital, Phulbari-11, Pokhara, Nepal; 3 Social and Administrative Pharmacy, Clinical Pharmacy and Practice Section, College of Pharmacy, Qatar University, Doha, Qatar; 4 Department of Pharmacology, American University of the Caribbean School of Medicine, University Drive at Jordan Road, Cupecoy, St. Maarten; 5 Department of Pharmacy Practice, College of Pharmacy, Gulf Medical University, Ajman, United Arab Emirates

**Keywords:** Availability, Rationality, Fixed-dose drug combinations, Nepal

## Abstract

**Background:**

The medications that are registered and available in a country are meant for the prevention and treatment of ailments and diseases. However, a lack of effective regulatory bodies and operative control mechanisms, especially in developing countries, promotes irrational and inappropriate use of medicines. This study aims to evaluate the availability and rationality of unregistered fixed-dose drug combinations (FDCs) in Nepal.

**Methods:**

A snowball sampling method with visits to 20 retail pharmacies in each of five major cities in Nepal was used to assess the availability of unregistered FDCs. To justify the rationality of the FDCs obtained from these five cities, the toolkit developed by Health Action International Asia-Pacific (HAI-AP) was used.

**Results:**

Altogether, 41 unregistered FDCs were obtained from the five cities. Among the total 41 FDCs, a majority were anti-inflammatory/analgesic/antipyretics. A maximum of eight drugs and a minimum of two drugs per combination were present among the total 41 FDCs, with a majority in the form of tablets followed by suspensions. The cost ranges from a minimum of 3.7 Nepalese Rupees (NRs) (= USD 0.05) to a maximum of 240 NRs (= USD 3.15). None of the FDCs fulfilled all the fundamental requirements as stated in the toolkit; thus, they were categorized as ‘irrational’.

**Conclusions:**

Unregistered FDCs are available in the Nepalese pharmaceutical market. All the unregistered FDCs found in our study were ‘irrational’ as per the HAI-AP toolkit. Regulatory authorities should initiate strict monitoring and appropriate regulatory mechanisms to prohibit the use of unregistered and irrational FDCs.

## Background

In general, all the formulations that are registered and available in a country are meant for the prevention and treatment of ailments and diseases [1]. In Nepal, several fixed-dose drug combinations (FDCs) are available in the market, many of them being irrational [2]. Irrational FDCs have several disadvantages, e.g., wastefulness, unnecessary additional cost, the difficulty of adjusting the dose, increased risk of adverse drug reactions, and uncertainty about the adequacy of the amount of a medicine in the combinations. In general, combinations of two or more active drugs in a single dosage form are classified as FDCs [3]. FDCs are available in Nepal for the treatment of various ailments ranging from nutritional deficiencies to cardiovascular diseases, but usually their rationality is doubtful. Meanwhile, there are no guidelines or criteria to identify the rationality of the combination. Health Action International Asia-Pacific (HAI-AP), a part of an independent global network, has worked to increase access to essential medicines and improve their rational use. The organization developed a toolkit to identify the rationality of FDCs based on 15 requirements or criteria developed based on the available data and scientific and medical principles [4]. This toolkit has been used by researchers in India [5] and pilot tested in Nepal investigating the registration status, availability and utilization pattern of FDCs in the Western region of Nepal and reported the presence FDCs on the market that are not registered with the national drug regulatory authority of Nepal [6]. FDCs are also widely used in different health care settings. Similarly, a preliminary study done in six major cities of Nepal identified the existence of irrational fixed-dose drug combinations (IFDCs) and suggested the need for immediate regulatory measures to control their availability in the country [2].

Since many FDCs are available in the Nepalese pharmaceutical market, it is difficult to determine whether a particular combination is rational. Many such FDCs are not even registered on the drug list of Nepal but are nonetheless available in the market and can be purchased without necessarily presenting a valid prescription. In developing countries such as Nepal and India, there is a widespread distribution and availability of IFDCs, but essential medicines are scarce. [1, 7]. Medical experts worldwide have expressed serious concerns over the increased marketing of FDCs by pharmaceutical companies, particularly in developing countries [8]. Studies from Nepal also document significant use of IFDCs and other combinations of doubtful efficacy [6, 9, 10].

The Current Index of Medical Specialties (CIMS) and Monthly Index of Medical Specialties (MIMS) list more than 100 IFDCs that are not approved in any developed countries but are being marketed in developing countries such as Nepal and India [11]. Nepal shares an open border with India, where there is an intense use of FDCs, which poses an additional risk [12]. Many FDCs available in Nepal are of Indian origin. Although the regulatory requirements for the approval of combination products vary across countries, there are no such specific regulations in Nepal. There are no clear guidelines or criteria to suggest whether the particular FDC is rational or irrational. Moreover, the registered drug list of Nepal is not periodically evaluated. In addition, there are no guidelines for the manufacturing and marketing of FDCs. The findings of this study would provide evidence to the relevant drug regulatory authority about the scenario of rampant availability and utilization of IFDCs in Nepal. This study is also expected to provide evidence on the existence of products in the Nepalese market and the extent of the potential problems the products could cause to the public. Despite the unavailability of clear and concise guidelines, the toolkit used in the study may act as a current evidence-based method or a guiding principle to classify an FDC as either ‘rational’ or ‘irrational’.

This study aimed to assess the availability and to justify the rationality of unregistered fixed-dose drug combinations in five major cities in Nepal.

## Methods

### Study design

This study applied a market survey method to evaluate the availability of unregistered FDCs in five major cities of Nepal.

### Study setting

The market survey was carried out in five major cities in Nepal. These five cities were selected because they are the major cities in Nepal in terms of population, area and economy [13]. The details on these cities are shown below:

- *Kathmandu*: Kathmandu is the capital and the largest metropolitan city of Nepal, with a population of approximately 1.183 million. Tourism is a major source of income for much of the city's population.
- *Biratnagar*: Biratnagar is Nepal’s second biggest city and is located in Koshi Zone on the southern plain land belt of Nepal, near the southeastern border with India. The city is only three kilometers away from the Indian border. The population of the municipality is 201,125.
- *Birgunj*: Birgunj is a sub-metropolitan municipality and border town in Parsa District in the Narayani Zone of southern Nepal. It lies 190 km west of the capital, Kathmandu, and two kilometers north of the border of the Indian state of Bihar. The population of Birgunj is approximately 300,000. As an entry point to Nepal from the Indian cities of Patna and Calcutta, it is also known as the gateway to Nepal.
- *Bhairahawa*: Bhairahawa (now called Siddharthanagar) is a town and municipality located in Rupandehi District in Lumbini Zone of southern Nepal, near the southwestern border with India. The city is only five kilometers away from India. The population of this city is approximately 163,483. Being located near the border of India and Nepal, it plays an important role in the import and export business.
- *Pokhara*: Pokhara is the third largest city in Nepal after Kathmandu and Biratnagar. It is the headquarter of Kaski District, Gandaki Zone and the Western Development Region. It has a population of approximately 255,465 people.

### Study population and sampling

A total of 20 retail pharmacies from each city were visited to assess the availability of unregistered FDCs. To find pharmacies selling unregistered FDCs, we used snowball sampling, a non-probability sampling method used when there is no sampling frame and the desired sample characteristics are rare. In this method, also known as referral or chain-referral sampling, a subject refers the investigator to other subjects who fit the study requirements [14]. Chain-referral sampling of a hidden population begins with a convenience sample of initial subjects that serve as ‘seeds’ through which other subjects are recruited. We were able to recruit 20 pharmacies from each city to find unregistered FDCs.

#### Inclusion and exclusion criteria

All the available FDCs that are not registered in Nepal were included in the study. The unregistered FDCs found in pharmacies from different cities were purchased. Identical FDCs of different brands were excluded from the study. Topical and intravenous FDCs were excluded from the study.

### Study tools

The following tools were used in the study:

#### HAI-AP toolkit to identify the rationality of fixed-dose drug combinations

As a goal to improve the rational use of medicines, HAI-AP initiated an advocacy program and a campaign to remove irrational FDCs. The toolkit developed by HAI-AP is used in this study to identify the irrational FDCs (Appendix 1) [4]. The literature basis for HAI-AP advocacy and its campaign to remove irrational FDCs is based on the 39^th^ Report, 2005, compiled by the World Health Organization (WHO) Expert Committee on Specifications for Pharmaceutical Preparations. The report consists of necessary criteria for developing new FDCs for the countries that do not yet have guidelines for FDCs [15]. The toolkit was pilot tested in Nepal and applied in this research [6].

#### International Network for Rational Use of Drugs (INRUD) indicators

The INRUD indicators are intended to measure aspects of health provider behavior in health care facilities in a reliable way, irrespective of who collects the data [16]. The indicators provide information to health care managers concerning medicine use, prescribing habits and important aspects of patient care. Since, behavior in health care facilities was not analyzed in the study, the indicators were modified according to the study objectives. Parameters such as ‘drug containing herbal constituents’ and ‘drugs containing vitamins and minerals’ were added to the indicators (Appendix 2).

### Data analysis procedure

The FDCs available from different cities were analyzed as per the study objectives. The SPSS program, version 16.0 (SPSS Inc. Released 2007. SPSS for Windows, Version 16.0. Chicago, SPSS Inc.), was used to compute the descriptive statistics.

A toolkit developed by HAI-AP was used to assess the rationality of each FDC. Before the toolkit was applied, all of the FDCs obtained from different cities were classified into different scenarios as per the HAI-AP toolkit. The various scenarios are described below:

(i) Scenario 1: The new FDC contains the same active ingredients in the same doses as an existing FDC. It is a ‘generic’ of the existing FDC: they are ‘multisource’ products. The quality, safety and efficacy of the existing product have been established.
(ii) Scenario 2: The new FDC contains the same active ingredients in the same dose as an established regimen of single-entity products, and the dosage regimen is the same. Alternatively, the established regimen may involve combinations of single entities and FDCs, for example, a single entity combined with an FDC that contains two active ingredients.
(iii) Scenario 3: The FDC combines active ingredients that are of established safety and efficacy but have not previously been used in combination for this indication. The new FDC comprises a combination for which safety and efficacy have been established, but that will be used in a different dosage regimen.
(iv) Scenario 4: The FDC contains one or more new chemical entities.
(v) Scenario 5: FDC containing two or more drugs with uncertain safety and efficacy.

## Results

### Part I: Analysis of the unregistered fixed-dose drug combinations in Nepal

Altogether, 41 FDCs were obtained from the five major cities of Nepal. The details from the cities are shown in Table 1 for Biratnagar, Table 2 for Birgunj, Table 3 for Bhairahawa, Table 4 for Pokhara and Table 5 for Kathmandu.

**Table 1:** Fixed-dose drug combinations available in Biratnagar (*n* = 9)

S. No	Ingredients	Therapeutic class	Dose	Formulation	Price in Nepalese rupees
A	Furazolidone 25 mg + Metronidazole 75 mg	Antidiarrhoeals/GI anti-infectives	Both	Suspension	13.7
B	Oxetacaine 10 mg + Aluminium Hcl 0.291 + Magnesium Hydroxide 98 mg	Drugs acting on gastrointestinal tract	Adults	Suspension	110.1
C	Amoxycillin trihydrate 125 mg + Bromhexine Hcl 4 mg	Antibacterials	Children	Syrup	41.6
D	Norfloxacin 400 mg + Tinidazole 600 mg + Lactic Acid Bacillus 120 million spores	Antidiarrhoeals/GI anti-infectives	Adults	Tablet	106.8
E	Ofloxacin 100 mg + Metronidazole 200 mg	Antidiarrhoeals/GI anti-infectives	Adults	Suspension	54.4
F	Prophenazone 150 mg + Paracetamol 250 mg + Caffeine 50 mg	Anti-inflammatory/Analgesic/Antipyretic	Adults	Tablet	20.0
G	Paracetamol 500 mg + Serratidopeptidase 15 mg + Aceclofenac 100 mg	Anti-inflammatory/Analgesic/Antipyretic	Adults	Tablet	189.2
H	Nimesulide 100 mg + Paracetamol 500 mg + Cetrizine 5 mg + Phenylpropanalamine 25 mg + Caffeine 25 mg	Cough and cold remedies	Adults	Tablet	69.8
I	Multivitamin combination + Oxytetracycline 250 mg	Antibacterials	Adults	Capsule	10.0

1 US $ = Approximately 76 NRs

**Table 2:** Fixed-dose drug combinations available in Birgunj (*n* = 11)

S. No	Ingredients	Therapeutic class	Dose	Formulation	Price in Nepalese rupees
A	Furazolidone 100 mg + Metronidazole 300 mg	Antidiarrhoeals/ GI anti-infectives	Adults	Tablet	85.8
B	Paracetamol 500 mg + Pseudoephedrine 32 mg + Cetrizine 10 mg	Cough and cold remedies	Adults	Tablet	32.0
C	Paracetamol 500 mg + Pseudroephedrine 30 mg + Cetrizine 30 mg	Cough and cold remedies	Adults	Tablet	25.0
D	Paracetamol 500 mg + Phenylpropanalamine 25 mg + Caffeine 32 mg	Anti-inflammatory/Analgesic/Antipyretic	Adults	Tablet	33.6
E	Mefenamic acid 250 mg + Dicyclomine HCl 10 mg	Antispasmodics	Both	Tablet	32.0
F	Norfloxacin 400 mg + Tinidazole 600 mg + Betacyclodextrin	Antidiarrhoeals/ GI anti-infectives	Adults	Tablet	55.3
G	Ofloxacin 50 mg + Metronidazole 120 mg	Antibacterials	Both	Suspension	48.4
H	Norfloxacin 400 mg + Metronidazole 500 mg	Antibacterials	Adults	Tablet	45.4
I	Ofloxacin 50 mg + Tinidazole 75 mg	Antibacterials	Both	Tablet	92.5
J	Paracetamol 500 mg + Dicyclomine 20 mg	Anti-inflammatory/Analgesic/Antipyretic	Adults	Tablet	19.7
K	Ofloxacin 50 mg + Ornidazole 125 mg	Antibacterials	Adults	Tablet	34.0

1 US $ = Approximately 76 NRs

**Table 3:** Fixed-dose drug combinations available in Bhairahawa (*n* = 13)

S. No	Ingredients	Therapeutic class	Dose	Formulation	Price in Nepalese rupees
A	Metronidazole 100 mg + Furazolidone 30 mg	Antidiarrhoeals/GI anti-infectives	Both	Suspension	88.0
B	Ciprofloxacin 500 mg + Ornidazole 500 mg	Antibacterial	Adults	Tablet	100.0
C	Cinnarizine 20 mg + Domperidone 15 mg	Antiemetic	Adults	Tablet	180.0
D	Dextromethorphan 10 mg + Phenylepherine 5 mg + Chlorpheniramine Maleate 2 mg	Cough and cold remedies	Both	Syrup	156.0
E	Ibuprofen 400 mg + Paracetamol 325 mg + Chlorzoxazone 250 mg	Anti-inflammatory/Analgesic/Antipyretic	Adults	Tablet	40.0
F	Ofloxacin 200 mg + Ornidazole 500 mg + Lactic Acid Bacillus 60 million spores	Antibacterial	Adults	Tablet	156.0
G	Aluminium hydroxide 125 mg + Magnesium hydroxide 125 mg + Simethicone 25 mg + Lignocaine 5 mg	Drugs acting on gastrointestinal tract	Both	Suspension	87.0
H	Nimesulide 50 mg + Paracetamol 125 mg	Anti-inflammatory/Analgesic/Antipyretic	Children	Suspension	77.0
I	Aceclofenac 100 mg + Paracetamol 500 mg + Serratidopeptidase 15 mg	Anti-inflammatory/Analgesic/Antipyretic	Adults	Tablet	155.0
J	Metronidazole 100 mg + Norfloxacin 100 mg	Antidiarrhoeals/Gastro Intestinal anti-infectives	Children	Suspension	60.0
K	Ofloxacin 50 mg + Tinidazole 100 mg	Antibacterials	Children	Suspension	130.0
L	Ofloxacin 200 mg + Ornidazole 500 mg	Antibacterials	Adults	Tablet	190.0
M	Pancreatin 170 mg + Oxbile extract 50 mg + Ginger oleoresin 2 mg + Activated charcoal 50 mg	Gastrointestinal agents	Adults	Tablet	46.7

1 US $ = Approximately 76 NRs

**Table 4:** Fixed-dose drug combinations available in Pokhara (*n* = 4)

S. No	Ingredients	Therapeutic class	Dose	Formulation	Price in Nepalese rupees
A	Diclofenac Sodium 50 mg + Chlorzoxazone 250 mg + Paracetamol 325 mg	Anti-inflammatory/Analgesic/Antipyretics	Adults	Tablet	20.0
B	Diphenoxylate Hcl 2.5 mg + Atropine Sulphate 0.025 mg + Furazolidone 50 mg	Antidiarrhoeals/ GI anti-infectives	Adults	Capsule	3.6
C	Norfloxacin IP 400 mg + Tinidazole 600 mg	Antidiarrhoeals/ GI anti-infectives	Adults	Tablet	240.0
D	Diclofenac Sodium 50 mg + Paracetamol 500 mg + Chlorpheniramine Maleate 4 mg + Pseudoephedrine	Anti-inflammatory/Analgesic/Antipyretics	Adults	Tablet	24.3

1 US $ = Approximately 76 NRs

**Table 5:** Fixed-dose drug combinations available in Kathmandu (*n* = 4)

S. No	Ingredients	Therapeutic class	Dose	Formulation	Price in Nepalese rupees
A	Loratidine 5 mg + Ambroxyl HCl 30 mg + Guaiphensin 50 mg	Cough and cold remedies	Children	Syrup	60.4
B	Pantoprazole 40 mg + Domperidone 10 mg	Drugs acting on gastrointestinal tract	Adults	Tablet	183.0
C	Diclofenac Sodium 50 mg + Paracetamol 500 mg + Chlorpheniramine Maleate 4 mg + Magnesium Trisilicate 100 mg	Anti-inflammatory/Analgesic/Antipyretic	Adults	Tablet	80.0
D	Paracetamol 500 mg + Serratidopeptidase 10 mg + Diclofenac 50 mg	Anti-inflammatory/Analgesic/Antipyretic	Adults	Tablet	55.0

1 US $ = Approximately 76 NRs

#### Comparison of retail price of similar fixed-dose drug combinations between cities

Among the total 41 FDCs, only 7 FDCs were similar and could be found in multiple cities. The details are shown in Table 6.

**Table 6:** Comparison of retail prices (NR) of similar FDCs between cities

Biratnagar	Birgunj	Bhairahawa	Pokhara	Kathmandu
(Furazolidone 25 mg + Metronidazole 75 mg)	(Furazolidone 25 mg + Metronidazole 75 mg)	na	na	na
Suspension	Tablet			
NRs 13.7	NRs 85.8			
(Norfloxacin 400 mg + Tinidazole 600 mg + + Betacyclo-dextrin)	(Norfloxacin 400 mg + Tinidazole 600 mg + Betacyclo-dextrin)	na	(Norfloxacin IP 400 mg + Tinidazole 600 mg)	na
Tablet	Tablet		Tablet	
NRs 106.8	NRs 55.3		NRs 240.0	
(Paracetamol 500 mg + Serratidopeptidase 15 mg + Aceclofenac 100 mg)	na	(Aceclofenac 100 mg + Paracetamol 500 mg + Serratidopeptidase 15 mg)	na	na
Tablet		Tablet		
NRs 189.2		NRs 155.0		

*na* not available

#### Comparison of number of ingredients in fixed-dose drug combinations based on retail price

Among the FDCs sampled, a maximum of eight (multivitamin combination with antibacterial) to a minimum of two (antibacterial) single-entity products were present in a single FDC. The details on the number of ingredients in FDCs are shown in Table 7.

**Table 7:** Comparison of number of ingredients in fixed-dose drug combinations (*n* = 41)

No of ingredients in FDC	N	Percentage
2	18	43.9
3	18	43.9
4	3	7.4
5	1	2.4
8	1	2.4
Total	41	100

#### Classification of fixed-dose drug combinations based on INRUD indicators and other criteria

Among all FDCs found in five major cities of Nepal, only one drug, ‘furazolidone’, was found in a combination that falls under the United Nations (UN) ‘Consolidated list of products whose consumption and/or sale have been banned, withdrawn, severely restricted or not approved by governments’. Similarly, one FDC containing ‘ginger oleoresin’ as an herbal constituent and one FDC containing a ‘multivitamin combination’ with antibacterials were found. The details of the study are shown in Table 8.

**Table 8:** Classification of fixed-dose drug combinations based on some of the INRUD indicators and other criteria (*n* = 41)

Indicators	Frequency	Percentage
Drugs with injectable preparations	-	-
Drugs that falls under UN “Consolidated list of products whose consumption and/or sale have been banned, withdrawn, severely restricted or not approved by Governments	1	2.4
Drugs from Nepalese National Formulary (NNF), 1997	-	-
Drugs from Essential Drug List of Nepal (3^rd^ Revision, 2002)	-	-
Drug containing herbal constituents	1	2.4
Drugs containing vitamins and minerals	1	2.4

‘-’Not available

### Part II: Application of a toolkit developed by HAI-AP

HAI-AP developed a toolkit to identify IFDCs. The study was carried out in two phases:

- **Phase I**: Classification of FDCs found from different cities into different scenarios, and
- **Phase II**: Application of a toolkit to identify IFDCs.

#### Phase I: Classification of fixed-dose drug combinations into different scenarios

Each FDC obtained from any of the five cities was classified into five different scenarios that comprise a set of requirements for marketing authorization of an FDC. A total of 41 FDCs from the five cities under study were classified according to different scenarios. The details are shown in Table 9.

**Table 9:** Classification of drugs according to different scenarios

Scenarios	Drugs
Scenario 1	-
Scenario 2	• Nimesulide BP 50mg + Paracetamol IP 125 mg
Scenario 3	• Paracetamol IP 500 mg + Serratidopeptidase 15 mg + Aceclofenac IP 100 mg • Propylphenazone IP 150 mg + Paracetamol IP 250 mg + Caffeine IP 50 mg • Nimesulide BP 100 mg + Paracetamol IP 500 mg + Cetrizine HCl BP 5 mg + Phenylpropanolamine Hcl BP 25 mg + Caffeine IP 25 mg • Oxetacaine BP 10 mg + Aluminium HCl 0.291 g + Magnesium Hydroxide IP 98 mg • Oxytetracycline HCl IP 250 mg + Thiamine mononitrate IP 2.5 mg + Riboflavin IP 2.5 mg + Niaciamide IP 25 mg + Pyridoxine HCl IP 0.5 mg + Calcium panthothenate IP 5 mg + Vitamin B12 IP 3 mcg + Folic acid IP 0.375 mg • Mefenamic Acid IP 250 mg + Dicyclomine HCl IP 10 mg • Paracetamol IP 500 mg + Phenylpropanolamine HCl BP 25 mg + Caffeine Anhydrous IP 32 mg • Paracetamol IP 500 mg + Pseudoephedrine HCl IP 32 mg + Cetrizine Dihydrochloride BP 10 mg
	• Paracetamol IP 500 mg + Pseudoephedrine HCl IP 30 mg + Cetrizine Dihydrochloride IP 30 mg • Paracetamol IP 500 mg + Dicyclomine HCl IP 20 mg • Ibuprofen IP 400 mg + Paracetamol IP 325 mg + Chlorzoxazone USP 250 mg • Aceclofenac BP 100 mg + Paracetamol IP 500 mg + Serratidopeptidase 15 mg • Aluminium hydroxide gel IP 125 mg + Magnesium hydroxide IP 125 mg + Simethicone IP 25 mg + Lignocaine HCl IP 5 mg • Dextromethorphan Hydrobromide IP 10 mg + Phenylephrine HCl IP 5 mg + Chlorpheniramine Maleate IP 2 mg • Diclofenac Sodium IP 50 mg + Paracetamol IP 500 mg + Chlorpheniramine Maleate IP 4 mg + Magnesium Trisilicate IP 100 mg • Diclofenac Sodium IP 50 mg + Chlorzoxazone USP 250 mg + Paracetamol IP 325 mg • Diphenoxylate Hcl IP 2.5 mg + Atropine Sulphate 0.025 mg + Furazolidone 50 mg • Loratidine USP 5 mg + Ambroxol HCl BP 30 mg + Guaifenesin IP 50 mg • Paracetamol IP 500 mg + Serratidopeptidase 10 mg + Diclofenac Potassium BP 50 mg • Diclofenac Sodium IP 50 mg + Paracetamol IP 500 mg + Chlorpheniramine Maleate IP 4 mg + Magnesium Trisilicate IP 100 mg • Pantoprazole 40 mg + Domperidone BP 10 mg • Cinnarizine BP 20 mg + Domperidone maleate BP 15 mg
Scenario 4	• Amoxycillin Trihydrate IP 125 mg + Bromhexine HCl IP 4 mg • Norfloxacin IP 400 mg + Tinidazole IP 600 mg + Betacyclodextrin • Pancreatin IP 170 mg + Oxbile extract 50 mg + Ginger oleoresin 2 mg + Activated charcoal IP 50 mg
Scenario 5	• Furazolidone IP 25 mg + Metronidazole Benzoate IP 75 mg • Ofloxacin IP 100 mg + Metronidazole Benzoate IP 200 mg • Norfloxacin IP 400 mg + Tinidazole IP 600 mg + Lactic Acid Bacillus 120 million spores • Ofloxacin IP 200 mg + Ornidazole 500 mg • Furazolidone IP 100 mg + Metronidazole IP 300 mg • Norfloxacin IP 400 mg + Metronidazole IP 500 mg • Ofloxacin USP 50 mg + Metronidazole Benzoate IP 120 mg • Ofloxacin IP 50 mg + Ornidazole 125 mg • Ciprofloxacin HCl IP 500 mg + Ornidazole 500 mg • Ofloxacin USP 200 mg + Ornidazole 500 mg • Ofloxacin USP 200 mg + Ornidazole 500 mg + Lactic Acid Bacillus 60 million spores • Metronidazole Benzoate 100 mg + Furazolidone IP 30 mg • Metronidazole benzoate 100 mg + Norfloxacin IP 100 mg • Ofloxacin USP 50 mg + Tinidazole IP 100 mg • Norfloxacin IP 400 mg + Tinidazole IP 600 mg + Lactic Acid Bacillus 60 msp

#### Phase II: Application of a HAI-AP toolkit

Among the 41 FDCs found from five major cities in Nepal, none of the FDCs fulfilled all the necessary requirements mentioned in the toolkit. The toolkit states 15 parameters that need to be fulfilled by any given FDC. All FDCs fulfilled three or fewer criteria. Certain criteria such as bioavailability data, bioequivalence data, preclinical pharmacology and safety are required only on some occasions, depending on the scenario. Details regarding the application of the toolkit for FDCs from different cities are shown in Table 10 for Biratnagar, Table 11 for Birgunj, Table 12 for Bhairahawa, Table 13 for Pokhara and Table 14 for Kathmandu.

**Table 10:** Application of toolkit to assess the rationality of fixed-dose drug combinations obtained from Biratnagar (*n* = 9)

Criteria	FDCs*								
	A	B	C	D	E	F	G	H	I
Rationale for the combination	✓	✓	✓	✓	✓	✓	✓	✓	✓
Balancing advantages and disadvantages of the combination	X	X	X	X	X	X	X	X	X
Marketing status in other countries	✓	✓	✓	✓	✓	✓	✓	✓	✓
Analysis of literature data in the submission	X	X	X	X	X	X	X	X	X
Pharmaceutical development studies	X	X	X	X	X	X	X	X	X
Good Manufacturing Practice (GMP) certification of sites of manufacture	X	X	X	X	X	X	X	X	X
A full quality data set	X	X	X	X	X	X	X	X	X
Bioavailability data	X	-	X	X	X	-	-	-	-
Bioequivalence data	X	-	-	X	X	-	-	-	-
Preclinical pharmacology and safety	X	-	X	X	X	-	-	-	-
Clinical safety and efficacy	X	X	X	X	X	X	X	X	X
Product information	✓	✓	✓	✓	✓	✓	✓	✓	✓
Plan for passive post marketing surveillance	X	X	X	X	X	X	X	X	X
Plan for active post marketing surveillance	X	X	X	X	X	X	X	X	X
Assurances	X	X	X	X	X	X	X	X	X

‘✓’ = Fulfilled, ‘**X**’ = Not fulfilled, ‘**-**’ = Not required

* **=** refer to Table 1

**Table 11:** Application of toolkit to assess the rationality of fixed-dose drug combinations obtained from Birgunj (*n* = 11)

Criteria	FDCs*										
	A	B	C	D	E	F	G	H	I	J	K
Rationale for the combination	✓	✓	✓	✓	✓	✓	✓	✓	✓	✓	✓
Balancing advantages and disadvantages of the combination	X	X	X	X	X	X	X	X	X	X	X
Marketing status in other countries	✓	✓	✓	✓	✓	✓	✓	✓	✓	✓	✓
Analysis of literature data in the submission	X	X	X	X	X	X	X	X	X	X	X
Pharmaceutical development studies	X	X	X	X	X	X	X	X	X	X	X
Good Manufacturing Practice (GMP) certification of sites of manufacture	X	X	X	X	X	X	X	X	X	X	X
A full quality data set	X	X	X	X	X	X	X	X	X	X	X
Bioavailability data	X	-	-	-	-	X	X	X	-	-	X
Bioequivalence data	X	-	-	-	-	-	X	X	-	-	X
Preclinical pharmacology and safety	X	-	-	-	-	X	X	X	-	-	X
Clinical safety and efficacy	X	X	X	X	X	X	X	X	X	X	X
Product information	✓	✓	✓	✓	✓	✓	✓	✓	✓	✓	✓
Plan for passive post marketing surveillance	X	X	X	X	X	X	X	X	X	X	X
Plan for active post marketing surveillance	X	X	X	X	X	X	X	X	X	X	X
Assurances	X	X	X	X	X	X	X	X	X	X	X

‘✓’ = Fulfilled, ‘**X**’ = Not fulfilled, ‘**-**’ = Not required * **=** refer to Table 2

**Table 12:** Application of toolkit to assess the rationality of fixed-dose drug combinations obtained from Bhairahawa (*n* = 13)

Criteria	FDCs*												
	A	B	C	D	E	F	G	H	I	J	K	L	M
Rationale for the combination	✓	✓	✓	✓	✓	✓	✓	✓	✓	✓	✓	✓	✓
Balancing advantages and disadvantages of the combination	X	X	X	X	X	X	X	X	X	X	X	X	X
Marketing status in other countries	✓	✓	✓	✓	✓	✓	✓	✓	✓	✓	✓	✓	✓
Analysis of literature data in the submission	X	X	X	X	X	X	X	X	X	X	X	X	X
Pharmaceutical development studies	X	X	X	X	X	X	X	X	X	X	X	X	X
Good Manufacturing Practice (GMP) certification of sites of manufacture	X	X	X	X	X	X	X	X	X	X	X	X	X
A full quality data set	X	X	X	X	X	X	X	X	X	X	X	X	X
Bioavailability data	X	X	-	-	-	X	-	-	-	X	X	X	X
Bioequivalence data	X	X	-	-	-	X	-	-	-	X	X	X	-
Preclinical pharmacology and safety	X	X	-	-	-	X	-	-	-	X	X	X	X
Clinical safety and efficacy	X	X	X	X	X	X	X	X	X	X	X	X	X
Product information	✓	✓	✓	✓	✓	✓	✓	✓	✓	✓	✓	✓	✓
Plan for passive post marketing surveillance	X	X	X	X	X	X	X	X	X	X	X	X	X
Plan for active post marketing surveillance	X	X	X	X	X	X	X	X	X	X	X	X	X
Assurances	X	X	X	X	X	X	X	X	X	X	X	X	X

‘✓’ = Fulfilled, ‘**X**’ = Not fulfilled, ‘**-**’ = Not required * **=** refer to Table 3

**Table 13:** Application of toolkit to assess the rationality of fixed-dose drug combinations obtained from Pokhara (*n* = 4)

Criteria	FDCs*			
	A	B	C	D
Rationale for the combination	✓	✓	✓	✓
Balancing advantages and disadvantages of the combination	X	X	X	X
Marketing status in other countries	✓	✓	✓	✓
Analysis of literature data in the submission	X	X	X	X
Pharmaceutical development studies	X	X	X	X
Good Manufacturing Practice (GMP) certification of sites of manufacture	X	X	X	X
A full quality data set	X	X	X	X
Bioavailability data	-	-	X	-
Bioequivalence data	-	-	X	-
Preclinical pharmacology and safety	-	-	X	-
Clinical safety and efficacy	X	X	X	X
Product information	✓	✓	✓	✓
Plan for passive post marketing surveillance	X	X	X	X
Plan for active post marketing surveillance	X	X	X	X
Assurances	X	X	X	X

‘✓’ = Fulfilled, ‘**X**’ = Not fulfilled, ‘**-**’ = Not required * **=** refer to Table 4

**Table 14:** Application of toolkit to assess the rationality of fixed-dose drug combinations obtained from Kathmandu (*n* = 4)

Criteria	FDCs*			
	A	B	C	D
Rationale for the combination	✓	✓	✓	✓
Balancing advantages and disadvantages of the combination	X	X	X	X
Marketing status in other countries	✓	✓	✓	✓
Analysis of literature data in the submission	X	X	X	X
Pharmaceutical development studies	X	X	X	X
Good Manufacturing Practice (GMP) certification of sites of manufacture	X	X	X	X
A full quality data set	X	X	X	X
Bioavailability data	-	-	-	-
Bioequivalence data	-	-	-	-
Preclinical pharmacology and safety	-	-	-	-
Clinical safety and efficacy	X	X	X	X
Product information	✓	✓	✓	✓
Plan for passive post marketing surveillance	X	X	X	X
Plan for active post marketing surveillance	X	X	X	X
Assurances	X	X	X	X

‘✓’ = Fulfilled, ‘**X**’ = Not fulfilled, ‘**-**’ = Not required * **=** refer to Table 5

## Discussion

This is the first study that evaluated the availability of unregistered FDCs in Nepal. The FDCs that were not registered with the drug control regulatory authority of Nepal were assessed. According to the WHO (1992), “fixed ratio combination products are acceptable only when the dosage of each ingredient meets the requirement of a defined population group and when the combination has a proven advantage over single compounds administered separately in therapeutic effect, safety or compliance” [17]. The study also attempted to test the toolkit developed by HAI-AP to identify an FDC either as ‘rational’ or ‘irrational’.

Altogether, 41 unregistered FDCs were found in five different cities of Nepal. Our findings are supported by a pilot conducted in six different cities of Nepal. The pilot study assessed the availability of FDCs that are not registered with the Department of Drug Administration (DDA) and are not approved by any standard drug information sources [2]. Nepal shares an open border with India. Thirty-three FDCs found in the study were from the plain lands (border area). The prevalence rate of IFDCs was higher in the cities that share an open border with India, and almost all IFDCs found were of Indian origin. On the other hand, a huge margin on IFDCs lures retail pharmacists to dispense and promote them. The booming availability of these FDCs in the market is also due to weak inspection or regulation by the relevant drug-regulating authority.

Among the total 41 FDCs, a majority were anti-inflammatory/analgesic/antipyretics. Analgesic, anti-inflammatory and antipyretic agents are used in various combinations in treating the common cold, influenza and inflammatory conditions. Many of these combinations have little documentary evidence that a preparation containing the combination is more effective than a single-ingredient preparation [18–20]. In addition, combining two NSAIDs may increase the side effects of each [1]. Nearly one-fourth of FDCs in our study were antibacterials, which in principle are ‘prescription-only’ medications but are easily available from retail pharmacies without a prescription in several parts of Nepal. The use of antibiotic FDCs can rapidly give rise to resistant strains of organisms, which is a matter of serious concern to the health care situation in our resource-poor country. A glaring example is the emergence of ciprofloxacin-resistant *Salmonella typhi* strains, which have made treatment of typhoid fever a difficult and expensive proposition today [3].

FDCs are becoming popular and are easily assessable for several reasons. They are easily available over the counter (OTC), provide immediate recovery, or are available at a low cost. To the average middle- or lower-middle-class family, an earlier recovery from an illness means an earlier return to work. The patient's objective is to get well early and get back to work. Pharmaceutical manufacturers of FDCs and prescribing doctors are aware of their patients’ psychology. Therefore, instead of spending the time needed to first perform a diagnostic test and then administer the appropriate medicine, they opt for prescribing FDCs. Administering FDCs is perceived as a benefit in terms of gain of workdays for the consumer. An FDC is more expensive, but since it involves only one visit to the physician, no test, and an immediate start to treatment resulting in a faster cure, it may be less expensive for the patient [19]. The knowledge and awareness regarding FDCs among prescribers are also crucial. In a study among dental clinician and residents reported that the study respondents have poor knowledge and lack of awareness about the advantages and disadvantages of FDCs [21].

A maximum of eight drug combinations and a minimum of two drug combinations were present among the total of 41 FDCs, with a majority in the form of tablets followed by suspension. The cost ranges from a minimum of 3.7 NRs to a maximum of 240 NRs. The cost advantage of FDCs compared to their individual components is still controversial, although some studies suggest that the cost of the combination is as high as three times the total added cost of individual component [22]. It is reported that prescribing combination therapy increases the cost of therapy [23].

The HAI-AP toolkit to identify IFDCs consists of fifteen parameters to be fulfilled by a particular FDC under study. The parameters in the toolkit state that the FDC should have a marketing status in different countries, clear ‘product information’ for the indicated diseases with information leaflets, a full high-quality data set, data for marketing authorization, well-characterized safety and efficacy including compliance with a suitable code of good manufacturing practice (GMP) during manufacture, etc. Such information on safety and effectiveness are crucial because study by Dalal et al. assessing the pharmacokinetic and pharmacodynamic of FDCs concluded that many of the FDCs approved were lacked of rationality [24]. In general, preclinical or clinical safety and efficacy data are not required. Similarly, FDCs from different cities were analyzed as per INRUD indicators. Other criteria were also taken into consideration, such as ‘drugs containing herbal constituents’, ‘drugs containing vitamins and minerals’, ‘presence of banned drugs in combination’, etc. INRUD has set up some criteria to design, test, and disseminate effective strategies to improve the way drugs are prescribed, dispensed, and used, with a particular emphasis on resource-poor countries [16].

The HAI-AP toolkit reported that all the 41 FDCs obtained from five different cities did not fulfill the necessary requirements or criteria set up in the toolkit. When a particular FDC does not fulfill the necessary data requirements for marketing authorization of an FDC in the relevant scenario, it is then labeled an ‘Irrational Fixed-Dose Combination’ that should be withdrawn from the market until its safety and efficacy are established. Similarly, INRUD indicators found that there were no injectable FDCs; the study found a combination of furazolidone and metronidazole that is banned in several countries because of concerns that it induces birth defects and cancer [25]. None of the FDCs were from NNF and EDL. One FDC each from Biratnagar and Bhairahawa had vitamin and herbal constituents, respectively. Usually, many vitamins are available as multivitamin combinations without any justification. Similarly, herbal remedies are often used in combination, usually in FDCs; however, the scientific literature supporting the efficacy of herbal therapies is incomplete [26]. Verifications of the potency and the therapeutic value of the herbal FDCs are very few [27].

### Limitations

The study had a few limitations. Although the study was able to find some FDCs in five major cities of Nepal, these do not represent the overall scenario. There might be many unregistered FDCs that we were not able to assess in the cities included in the study. Hence, the study cannot conclude that the FDCs found in this study represent the full complement of IFDCs in Nepal. The toolkit developed by HAI-AP was tested for the first time in our study. Although we sought collaboration with international experts, we were not able to obtain certain parameters such as bioavailability, bioequivalence data, clinical safety and efficacy. The limited literature and research in these areas made it difficult to interpret the findings conclusively. In contrast, this study’s methodology and findings could establish a good platform for future studies in other low- and middle-income countries.

### Recommendations

There is a need for extensive research in this area. There is also a need for an adequate awareness program for consumers regarding the hazards of IFDCs, along with careful monitoring and censoring of misleading claims by pharmaceutical industries. Moreover, there should be defined criteria to approve an FDC in the country. The use of the HAI-AP toolkit may be beneficial in these aspects. Regulatory authorities, healthcare professionals and pharmaceutical companies should join together to formulate an effective guideline for the FDCs. The regulatory approval process of FDCs need to be stringent because the failure for critical analysis of the scientific validity of the formulations will allow irrational and dangerous products to be sold freely in the market [28]. We recommend that the DDA, the drug regulatory authority of Nepal, initiate strict monitoring and appropriate regulatory initiatives to either ban or prohibit the use of IFDCs. Educational interventions for students as well as patients (consumers) at different levels may be required.

## Conclusions

Although the study had several limitations, we found that unregistered FDCs are available in the Nepalese pharmaceutical market. FDCs were available for treatment of different conditions, ranging from coughs and colds to bacterial infections, and in different dosage forms, from a maximum of eight to a minimum of two drugs. All the unregistered FDCs found in our study were ‘irrational’ as per the HAI-AP toolkit.

## Appendix 1

**Table 15:** Toolkit developed by HAI-AP to identify irrational FDCs

Requirements	Scenario 1	Scenario 2	Scenario 3	Scenario 4	Scenario 5
Rationale for the combination	Not usually	Not usually	✓	✓	✓
Balancing advantages and disadvantages of the combination	Not usually	Not usually	✓	✓	✓
Marketing status in other countries	✓	✓	✓	✓	✓
Analysis of literature data in the submission	Possibly for pharmaceutical development	Possibly for pharmaceutical development	✓	✓	✓
Pharmaceutical development studies	✓	✓	✓	✓	✓
Good Manufacturing Practice (GMP) certification of sites of manufacture	✓	✓	✓	✓	✓
A full quality data set	✓	✓	✓	✓	✓
Bioavailability data	Not usually	Not usually	Sometimes	✓	✓
Bioequivalence data	✓	✓	Sometimes	Sometimes	✓
Preclinical pharmacology and safety	Not usually	Not usually	Sometimes	✓	✓
Clinical safety and efficacy	Not usually	Not usually	✓	✓	✓
Product information	✓	✓	✓	✓	✓
Plan for passive post marketing surveillance	✓	✓	✓	✓	✓
Plan for active post marketing surveillance	Not usually	Not usually	✓	✓	✓
Assurances	✓	✓	✓	✓	✓

**✓** This is a requirement

## Appendix 2

**Table 16:** INRUD and other drug use indicators

Indicators	Frequency	Percentage
Drugs with injectable preparations		
Drugs that falls under UN ‘Consolidated list of products whose consumption and/or sale have been banned, withdrawn, severely restricted or not approved by Governments’		
Drugs from Nepalese National Formulary (NNF), 1997		
Drugs from Essential Drug List of Nepal (3^rd^ Revision, 2002)		
Drug containing herbal constituents		
Drugs containing vitamins and minerals		

## Abbreviations

CIMS: Current Index of Medical Specialties
DDA: Department of Drug Administration
EDL: Essential Drug List
FDCs: Fixed-dose Drug Combinations
GMP: Good Manufacturing Practice
HAI-AP: Health Action International Asia-Pacific
IFDCs: Irrational Fixed-dose Drug Combinations
INRUD: International Network for Rational Use of Drugs
MIMS: Monthly Index of Medical Specialties
NNF: Nepalese National Formulary
NSAIDs: Nonsteroidal Anti-inflammatory Drugs
NRs: Nepalese Rupees
OTC: Over-the-counter
SPSS: Statistical Package for the Social Sciences
USD: United States Dollar
WHO: World Health Organization
